# Defining the timing of respiratory syncytial virus (RSV) outbreaks: an epidemiological study

**DOI:** 10.1186/1471-2334-5-20

**Published:** 2005-03-31

**Authors:** Elena Terletskaia-Ladwig, Gisela Enders, Gunnar Schalasta, Martin Enders

**Affiliations:** 1 Institute for Virology, Infectiology and Epidemiology & Medical Diagnostic Laboratory Prof. G. Enders and Partners, Rosenbergstrasse 85, D-70 193, Stuttgart, Germany

## Abstract

**Background:**

Seasonal RSV infections occur every year and affect particularly children under six months of age. Passive immunoprophylaxis with monoclonal antibody Palivizumab is recommended in the period with high risk of RSV infection. This study aims to define the period for the southern part of Germany (Stuttgart area).

**Methods:**

Epidemiological analysis of the RSV situation in southern Germany from 1996 to 2004 and comparison of results with literature was made. The respiratory tract specimens were sent in for the detection of RSV mainly by paediatric clinics. Detection of RSV was carried out mainly by real-time RT-PCR or by ELISA "Pathfinder". RSV outbreaks were depicted as an absolute number and as a percentage of RSV diagnoses in a month. Onsets, offsets, peaks, duration and severity of RSV seasons were defined and analysed.

**Results:**

An early season with strong RSV activity (early-high phase) was followed by a weaker late season (late-low phase) in a regular biennial rhythm. However, onsets, offsets and durations of outbreaks varied significantly from year to year. RSV epidemics in southern Germany were found to oscillate in an antiphase with RSV epidemics in Finland and Sweden.

**Conclusion:**

The long-term regular biennial rhythm allows predicting whether the next outbreak will be late or early and whether RSV activity will be strong or weak. Not foreseeable, however, is the precise time of increase and decrease of RSV activity. Moreover, the regular seasonal pattern may be disrupted by irregular outbreaks. Thus, activity of RSV has to be monitored every year to define the period with high risk of infection.

## Background

Respiratory syncytial virus (RSV) is the most common cause of viral bronchiolitis and pneumonia in infants and children under two years of age. Premature infants, children with chronic lung diseases (e.g. bronchopulmonary dysplasia), children with congenital heart disease and immunodeficiency patients have high risk of a severe disease.

There are no efficient active vaccines and causal therapy, therefore prophylactic measures are especially important. Prospective surveillance of RSV infection and consequent implication of hygienic measures for preventing transmission and spread of RSV contribute effectively to reduction of hospital-acquired infections. Moreover, a humanized monoclonal antibody palivizumab (Synagis^®^, Abbott) was licensed in September 1999 in the European Union for passive immunoprophylaxis of RSV.

In view of the relatively low risk of RSV rehospitalisation among premature infants and high costs of immunoprophylaxis, additional individual risk factors and especially the local epidemiologic situation have to be considered in the case of Synagis^®^-administration [1-3].

RSV epidemics occur seasonally and last from four to six months. North of the equator the peak incidence of disease is observed from December to April [4-6]. In Europe, regular biennial patterns of RSV epidemics were described in which a weaker late season was followed by a more severe early season [7-11]. The discovery of a regular pattern of RSV epidemics seemed to allow the prediction of outbreaks.

We characterized RSV epidemics in southern Germany with a focus on the Stuttgart area – onset, offset, peak, intensity, duration of outbreaks and seasonal rhythm – and compared them with already published data. The aim of this work was to use obtained data to fix a time limit for Synagis^® ^prophylaxis by precisely determining the period with high risk of RSV.

## Methods

### Samples

Nasopharyngeal samples were sent from paediatric hospitals and practitioners from children with suspected RSV infection. Samples were sent cooled in transport medium Veal Infusion Broth (BD Difco™, USA).

### Detection of RSV

From January 1996 to April 2001 the detection of RSV was carried out by RT-PCR and ELISA Pathfinder (Bio-Rad, USA). 70% of samples were tested by both methods, 20% of samples were tested only by ELISA and 10% only by RT-PCR. Since May 2001 only RT-PCR has been used for RSV testing because of its higher sensitivity and specificity.

### Extraction of Nucleic Acid (NA)

From January 1996 up to September 2001 NA extraction was performed manually using the High Pure Viral Nucleic Acid Kit (Roche Diagnostics GmbH, Mannheim, Germany). Briefly: Virus lysis is performed by incubation of the samples in a lysis/binding buffer in the presence of proteinase K and poly (A) carrier RNA. NA are bound to glass fibers and were subsequently recovered in 50 μl elution buffer by centrifugation through a glass fleece.

From September 2001 on NA extraction was performed on the MagNA Pure LC automated instrument (Roche Diagnostics GmbH, Mannheim, Germany) using the MagNA Pure LC Total Nucleic Acid Isolation Kit (Roche Diagnostics GmbH, Mannheim, Germany) for all preparations. The setting and preparation of the instrument were performed according to the manufacturer's instructions. Briefly, 200 μl of each sample was transferred to the sample cartridge and loaded onto the workstation together with the kit reagents. The instrument automatically performs all isolation and purification steps such as addition of lysis/binding buffer and magnetic glass particles (MGPs), binding of DNA to the MGPs, washing steps, elution of the pure NA (60 μl), and transfer of the NA to a cooled storage cartridge. NA extracted in this way is ready for use in RT-PCR applications.

### RT-PCR

A Dig-ELISA RT-PCR protocol was used from September 1996 up to October 1999 together with a commercial PCR-ELISA kit (Roche Diagnostics GmbH, Mannheim, Germany) for amplicon detection. A real-time RT-PCR protocol replaced the Dig-ELISA RT-PCR from October 1999 on.

For real-time RT-PCR, the LightCycler (LC) instrument (Roche Diagnostics GmbH, Mannheim, Germany) and primers and probes from the viral N gene, previously described by [12] were used. To enable rapid non-type specific simultaneous detection of both RSV subtypes, primer pairs A21/A102 for subtype A and B17/B120 for subtype B were used in combination with the probes APB48 for subtype A and BPB45 for subtype B in one reaction mixture. Probes contained the 5' reporter dye 6FAM and the 3' quencher dye TAMRA (TIB MOLBIOL, Berlin, Germany).

LC RT-PCR was performed in LC capillaries in a final reaction volume of 20 μl. Master mixes were based on the commercial QuantiTect™ Probe RT-PCR kit (QIAGEN, Hilden, Germany) supplemented with primers A21/A102/B17/B120 (f.c. 1,0 μM each), the probes APB48 and BPB45 (f.c. 0,25 μM each) and heat labile UNG (0,2 U) (Roche Diagnostics GmbH, Mannheim, Germany). Use of this kit allows both reverse transcription and PCR to take place in a single capillary, so there is no need to open the capillary once reverse transcription is accomplished. After pipetting of 13,2 μl of this reaction mixture into LC capillaries, 6,8 μl of the crude NA preparation was added. A 5 min. step at room temperature to enable UNG activity is followed by 20 min. of reverse transcription at 50°C. PCR is started with a 15 min. step at 95°C followed by 10 cycles of denaturation (95°C for 5 sec), annealing (with a 1°C decrease per cycle from 65°C to 55°C for 20 sec) and extension (72°C for 15 sec), followed by 40 cycles of denaturation (95°C for 0 sec), annealing (55°C for 20 sec) and extension (72°C for 30 sec).

### Epidemiological analysis

Epidemiological analysis included nine RSV seasons from January 1996 to June 2004. Only samples sent by hospitals and practitioners in southern Germany (Baden-Württemberg and Bavaria) were analyzed in this study. 80% of samples were sent from hospitals in the Stuttgart area. The following data were extracted from the laboratory database: laboratory identification number, age, sample withdrawal date, sender address and RSV antigen detection test results.

Onset, offset, peak and duration of outbreaks were determined for each year. In order to distinguish between the beginning/ending of an outbreak and background activity, the number of sporadic RSV cases in the summer months from June to October were calculated. A maximum of 5 cases in a month and 2 cases in a week were registered in this time. Therefore, onset month (week) of outbreak was defined as the first of two consecutive months (weeks) with at least 5(2) positive RSV findings. Offset month was defined as the last of the final two consecutive months (weeks) with at least 5(2) positive RSV findings. Duration was defined as the number of months (weeks) between the calculated onset and offset months (weeks) inclusive of those months (weeks). Outbreak peak was defined as the month (week) after onset with the highest number of positive test results.

Outbreaks were graphically depicted in two different forms: an epidemic curve by plotting the number of cases on the vertical axis and time on the horizontal axis and a graphic showing percentage of positive test results in a month.

The total number of positive RSV findings was considered for estimation of outbreak intensity.

## Results

Nasopharyngeal samples from a total of 3577 patients were tested in the period from January 1996 to June 2004. 32% of samples (1154) were found to be positive.

Our customers, paediatric hospitals and practitioners, who sent the samples, remained the same over the time period. 80% of samples were sent in by children's hospitals, 10% by practitioners and 10% by other private medical-diagnostic laboratories.

The age distribution of patients tested positive reflected the distribution for the whole material sent in: approximately 40% of RSV were infants younger than 3 months, 30% of those aged 3 to 6 months, 20% 7–12 months and 10 % 1 to 3 years. Patients older then 3 years were rarely tested (less then 1%).

From Januar 1996 to April 2001 a total of 1653 samples were tested both by EIA and RT-PCR. The sensitivity of EIA versus RT-PCR was 41% (112/273), specificity 98% (1358/1380).

**Figure 1:**
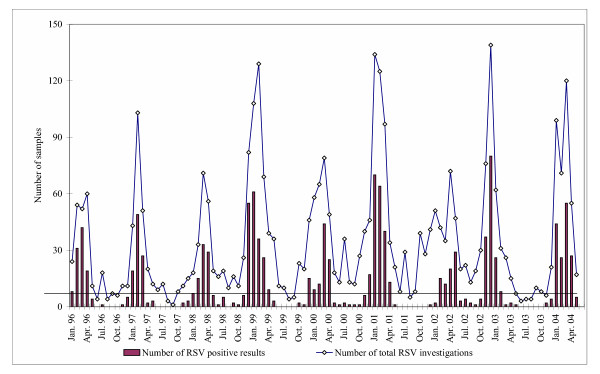
Absolute number of total RSV investigations and positive results by month, from January 1996 to May 2004.

The epidemic curve (Fig. 1) shows the seasonal distribution of acquired positive RSV results by month. The highest RSV activity was registered in the period from late autumn to spring. A clear biennial cycle of successive RSV outbreaks can be seen: Late weak seasons 1995/96, 1997/98, 1999/00, and 2001/02 (late-low phase, minor peaks) followed by more severe early seasons 1996/97, 1998/99, 2000/01 and 2002/03 (early-high phase, major peaks). Table 1 shows that the onsets, offsets and durations of outbreaks varied significantly from season to season. The late season can start relatively early in week 50 or relative late in week 6.

**Table 1:** Onset, peak, offset and duration (weeks) of RSV Outbreaks in Stuttgart, from 1996 to 2004.

Season	Onset	Peak	Offset	Duration
1995/1996 late season	1	12	17	17
1996/1997 early season	49	8	13	16
1997/1998 late season	2	13	20	19
1998/1999 early season	48	5	18	24
1999/2000 late season	50	14	19	23
2000/2001 early season	48	6	15	21
2001/2002 late season	6	20	23	18
2002/2003 early season	45	51	6	14
2003/2004 late season	1	14	19	19

**Figure 2:**
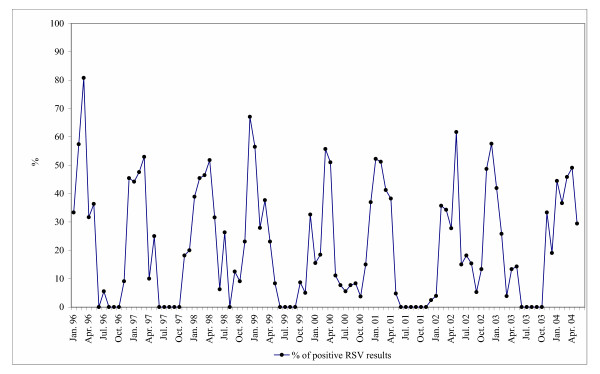
Percentage of RSV positive cases by month, from January 1996 to May 2004.

Fig. 2 presents the seasonality of RSV detection as a percentage of samples tested positive per month. Year-to-year alternation between late and early seasons can also be seen in Fig. 2 as in a Fig. 1. The monthly percentage of positive results in early-high seasons was the same as in late-low seasons or actually lower. Therefore any difference of the epidemics intensity cannot be established.

The comparison of seasonal rhythms with literature and surveillance data showed that RSV outbreaks in Stuttgart followed the same pattern as in Freiburg and in Switzerland. In Kiel the seasons from 1994/95 to 1996/97 started late. Since 1997/1998 the same 2-year cycle could be detected in Kiel as in Stuttgart and Freiburg. RSV epidemics in Finland and Sweden occurred synchronously with regular rhythm (high-early versus low-late phase), but were in antiphase with Germany RSV outbreaks (Table 2).

**Table 2:** Comparison of seasonal rhythm in European areas.

RSV Seasons	Finland [7]	Sweden [8]	Switzerland [11]	Germany, Freiburg [9]	Germany, Kiel [10,18]	Germany, Stuttgart
1982/1983	late					
1983/1984	early					
1984/1985	late	late				
1985/1986	early	early				
1986/1987	late	late				
1987/1988	early	early				
1988/1989	late	**late**	**early**			
1989/1990	early	**early**	**late**			
1990/1991		**late**	**early**	early		
1991/1992		**early**	**late**	late		
1992/1993		**late**	**early**	early		
1993/1994		**early**	**late**	late		
1994/1995			early	early	late	
1995/1996			late	late	late	
1996/1997			early	early	late	early
1997/1998			late		late	late
1998/1999			early		early	early
1999/2000			late		late	late
2000/2001					early	early
2001/2002					late	late
2002/2003					early	early
2003/2004					late	late

## Discussion

Age distribution analysis of our patients with diagnosed RSV infection confirmed once more that very small infants younger than three months have the highest risk of hospitalisation and therefore of being tested for RSV [9].

Sensitivity of EIA calculated versus RT-PCR was found to be very low, only 41 %. In the few studies in which Pathfinder RSV EIA was evaluated the sensitivity (up to100%) of this assay was calculated only in comparison with other less sensitive methods than PCR, such as cell culture, shell vial assay or direct immunofluorescence [13]. The low sensitivity of EIA in our study can also be explained with the instability of RSV, even though appropriate collection and transport conditions were assured. In addition, it must be considered that the nasal quantity of RSV correlates with disease severity in hospitalised infants [14]. In our study, the use of EIA did not affect the proportion of positives, since only a small number of samples were tested by this method only.

The discovered in Stuttgart long-term regular biennial rhythm allows prediction of whether the next outbreak will be late or early and whether RSV activity will be strong or weak. However, the value of this prediction is limited. The precise time of increase and decrease of RSV activity is not foreseeable (Table 1). Only local monitoring of RSV activity allows definition of the period with high risk of infection. Therefore, since November 2003 Laboratory Enders & Partner has published weekly from late autumn to spring the actual RSV epidemiological situation in the Stuttgart area on its website [15]. It is noted that our monitoring focused mainly on hospitalised children. With regard to RSV, this approach is acceptable while the proportion of RSV infections among inpatients is higher than in outpatients. However, a population-based approach used in the PRI.DE study may be more sensitive in the detection of offsets because it includes outpatients [16].

There is no generally accepted method for surveillance of RSV outbreaks. In order to compare the RSV epidemiological situation in Stuttgart with that in other areas, we investigated primarily which methods of RSV outbreak analysis are usually used.

In Europe, Waris [7] described nine RSV seasons. He ascertained the number of RSV laboratory diagnosis made at the Department of Virology, University of Turku, Finland per month in the time period from January 1981 to March 1990, and discovered a regular periodic biennial pattern of occurrence of RSV outbreaks. A weaker late season was followed by a more severe early season. Other similar epidemic curves were depicted by Reyes (seasons 1984–1993, Karolinska Institute, Sweden) [8], Berner (seasons 1988–1999, University of Freiburg, south Germany) [9], Weigl (seasons 1994–2001 University of Kiel, north Germany 2002) [10] and Duppenthaler (seasons 1988–2001, Switzerland) [11].

In the USA, the national Respiratory and Enteric Virus Surveillance System (NREVSS) monitors and summarizes the RSV surveillance results starting in July 1985 and publishes them on the NREVSS website [17]. The RSV activity in NREVSS reports is shown as median percent of positive for RSV results per month (week) and region. Onsets/offsets are defined as the first/last of two consecutive weeks (months) of at least 10% positive test results with at least two positive samples in the numerator of the reports of both weeks (months) [4,5].

In our study both methods of outbreak analysis were used. We acquired the number and percentage of positive cases per month.

Monitoring of RSV activity by calculation of the percentage of positive RSV detection in comparison to the number of positive cases has the advantage that the results show the real RSV situation independently of changes in population. Also, in our study, in outbreaks months at least 10% of samples were positive. However, in summer weeks, in which only few samples were sent in, the positive rate could be over 10% by only one positive sample. Therefore, it is also important to consider the number of sporadic cases in order to distinguish between the beginning/ending of an outbreak and background activity.

Onset, offset and peak months on the plot with the percentage curve were nearly the same as on the plot presenting the absolute number of positive cases. Early and late seasons were recognisable on our plot with the percentage curve. These fluctuations of RSV season onset weeks can also be observed in two Omaha Nebraska NREVSS surveillance laboratories [5]. It is noted that on the plot with percentage curve the intensity of epidemics cannot be seen. In seasons with low RSV intensity (small number of samples) the positive rate was as high (50–60%) as in seasons with high RSV activity. Therefore, this information is missing in NRVSS reports. Only the combination of both approaches to the monitoring of RSV outbreaks supplies complete information for the estimation of the epidemiological situation.

European surveillance centres present RSV data in different ways: The Pediatric Infectious Diseases Network on Acute Respiratory Tract Infections (PID-ARI.Net) Web&Warnsystem [18] (Kiel, Freiburg and Mainz, Germany) shows tables and incidence plot, the North Saxon State Health Office (NLGA) (Hanover, Germany) [19] uses percentage plot, the Swedish Institute for Infectious Disease Control (SMI) [20] presents tables, the Infectious Diseases Information System (ISIS) (the Netherlands) [21] shows different plots and tables. The use of different methods complicates the interpretation of data. As far as possible we compared seasonal RSV rhythm in Stuttgart with other European reports (Table 2).

This comparison showed that RSV seasonality in Stuttgart was in accordance with other areas of Germany and Switzerland [9-11,16,18]. The explanation for the absence of a regular pattern in Kiel before 1996 may be the low total number of samples tested and, therefore, the possibility of missed cases. The other possible explanation may be found by analyzing RSV epidemics in northern Europe. RSV epidemics in Finland and Sweden were also synchronized in time as outbreaks in Germany, but the early-high phase in northern Europe corresponded with late-low phase in southern Germany. Kiel is possibly located at the borderline between two areas with different rhythms [22].

Even in areas with a regular rhythm it may shift. Such a switchover can be observed in Chile. The figure depicted by Avendano, which presents RSV detection from 1989 to 2000, shows that after two cycles of late/early seasons the following season 1994 began repeatedly early, then the rhythm was resumed [23]. Also, in Gambia a regular pattern of outbreaks during 6 consecutive annual seasons was disrupted by 2 years of irregular outbreaks, followed by another 2 years of regular seasonal outbreaks [24].

## Conclusion

The long-term regular biennial rhythm allows predicting whether the next outbreak will be late or early and whether RSV activity will be strong or weak. Not foreseeable, however, is the precise time of increase and decrease of RSV activity. Thus, activity of RSV has to be monitored every year to define the period with high risk of infection. Moreover, different rhythms of epidemics in Europe and the theoretical possibility of their shift demonstrate that the present regionally limited monitoring is not sufficient. An RSV surveillance system in Europe similar to the existing influenza surveillance system is required. Analysis of spatio-temporal European data will contribute to the understanding of RSV epidemiology and predicting outbreaks. Correct identification of outbreaks will permit the limiting of the time period for Synagis^® ^administration and thus save costs.

## Competing interests

The author(s) declare that they have no competing interests.

## Authors' contributions

ET conceived, carried out the study and wrote the manuscript. GE supervised the study. GS carried out the assay development. ME conceived the study and critically reviewed the manuscript.

## Pre-publication history

The pre-publication history for this paper can be accessed here:



## References

[B1] Liese JG, Grill E, Fischer B, Roeckl-Wiedmann I, Carr D, Belohradsky BH (2003). Incidence and risk factors of respiratory syncytial virus-related hospitalizations in premature infants in Germany. Eur J Pediatr.

[B2] Roeckl-Wiedmann I, Liese JG, Grill E, Fischer B, Carr D, Belohradsky BH (2003). Economic evaluation of possible prevention of RSV-related hospitalizations in premature infants in Germany. Eur J Pediatr.

[B3] Deutsche Gesellschaft für Pädiatrische Infektiologie (DGPI). Prophylaxe von bedrohlichen RSV-Erkrankungen durch Palivizumab. http://www.dgpi.de/home/frame.html.

[B4] Gilchrist S, Torok TJ, Gary HE, Alexander JP, Anderson LJ (1994). National surveillance for respiratory syncytial virus, United States, 1985–1990. J Infect Dis.

[B5] Mullins JA, Lamonte AC, Bresee JS, Anderson LJ (2003). Substantial variability in community respiratory syncytial virus season timing. Pediatr Infect Dis J.

[B6] Simoes EA, Carbonell-Estrany X (2003). Impact of severe disease caused by respiratory syncytial virus in children living in developed countries. Pediatr Infect Dis J.

[B7] Waris M (1991). Pattern of respiratory syncytial virus epidemics in Finland: two-year cycles with alternating prevalence of groups A and B. J Infect Dis.

[B8] Reyes M, Eriksson M, Bennet R, Hedlund KO, Ehrnst A (1997). Regular pattern of respiratory syncytial virus and rotavirus infections and relation to weather in Stockholm, 1984 – 1993. Clin Microbiol Infect.

[B9] Berner R, Schwoerer F, Schumacher RF, Meder M, Forster J (2001). Community and nosocomially acquired respiratory syncytial virus infection in a German paediatric hospital from 1988 to 1999. Eur J Pediatr.

[B10] Weigl JA, Puppe W, Schmitt HJ (2002). Seasonality of respiratory syncytial virus-positive hospitalizations in children in Kiel, Germany, over a 7-year period. Infection.

[B11] Duppenthaler A, Gorgievski-Hrisoho M, Frey U, Aebi C (2003). Two-year periodicity of respiratory syncytial virus epidemics in Switzerland. Infection.

[B12] Hu A, Coltella M, Tam JS, Rappaport R, Cheng SM (2003). Simultaneous Detection, Subgrouping, and Quantitation of Respiratory Syncytial Virus A and B by Real-Time PCR. J Clin Microbiol.

[B13] Pedneault L, Robillard L, Turgeon JP (1994). Validation of respiratory syncytial virus enzyme immunoassay and shell vial assay results. J Clin Microbiol.

[B14] Buckingham SC, Bush AJ, Devincenzo JP (2000). Nasal quantity of respiratory syncytical virus correlates with disease severity in hospitalized infants. Pediatr Infect Dis J.

[B15] Labor Enders & Partner. Epidemiologische News. http://www.labor-enders.de/.

[B16] Forster J, Ihorst G, Rieger CH, Stephan V, Frank HD, Gurth H, Berner R, Rohwedder A, Werchau H, Schumacher M, Tsai T, Petersen G Prospective population-based study of viral lower respiratory tract infections in children under 3 years of age (the PRI.DE study). Eur J Pediatr.

[B17] The National Respiratory and Enteric Virus Surveillance System (NREVSS). Respiratory Syncytial Virus Trends. http://www.cdc.gov/ncidod/dvrd/revb/nrevss/rsvtre1.htm.

[B18] PID-ARI.Net. Web&Warnsystem. http://www.pid-ari.net/.

[B19] Niedersächsisches Landesgesundheitsamt (NLGA). http://www.nlga.niedersachsen.de/infekt/are_surveillance.htm.

[B20] Swedish Institute for Infectious Disease Control (SMI). RSV-rapporter.

[B21] The national Infectious Diseases Information System (ISIS), the Netherlands. http://www.rivm.nl/isis/lab/openbaar/phdz/index_88.html.

[B22] He D, Stone L Spatio-temporal synchronization of recurrent epidemics. Proc R Soc Lond B Biol Sci.

[B23] Avendano LF, Palomino MA, Larranaga C (2003). Surveillance for respiratory syncytial virus in infants hospitalized for acute lower respiratory infection in Chile (1989 to 2000). J Clin Microbiol.

[B24] van der, Sande MA, Goetghebuer T, Sanneh M, Whittle HC, Weber MW (2004). Seasonal variation in respiratory syncytial virus epidemics in the Gambia, West Africa. Pediatr Infect Dis J.

